# Crural Phlebitis

**Published:** 1851-01

**Authors:** David Hutchinson

**Affiliations:** Mooresville, Ind.


### THE NORTH-WESTERN
### MEDICAL AND SURGICAL JOURNAL.

VOL. III.] JANUARY, 1851. [No. 5.

### Part 1.—Original Communications.

### ARTICLE I.

*Cural Phlebitis, occurring in the Male.* By DAVID HUTCHINSON, M. D., of Mooresville, Ind.

Sept. 8th, 1850. James McNeff, *ætat* 12 years, large of his age, and of a sanguine constitution, was taken on the 6th, with chill, succeeded by high febrile action, which has continued without remission; pulse 120 per minute and sharp, though compressible; skin hot, tongue swollen, dry, cracked and red; complains of pain in the right hip and thigh, extending from the groin to the knee; the right foot is swollen; he says he cannot walk. Prescribed Calomel 12 grs., Morph. 1-2 gr., divided into 4 portions; to take a dose every four hours, to be followed by a dose of Castor Oil to act on the bowels.

9th. Fever all night: delirium, talks incessantly, frequent sharp pulse, 120 per minute, had two bilious looking evacuations from the bowels, tongue not quite so dry as yesterday, still swollen, coat yellow; still complains of pain in the groin and anterior part of the thigh; the inguinal region some swollen and very tender to the touch; thigh swelled. Prescribed 15 grs. Calomel, 1 1-2 grs. Ipecac, divided into five portions, one every three hours, to be followed by a dose of Castor Oil.

10th. Fever with delirium all night; slight remission of wo hours took place this morning, tongue coated, dry and cracked; pulse sharp, small and frequent; had two thin bilious evacuations from bowels; some swelling of inguinal glands, thigh swelled, hot and tender along the course of the femoral veins; pain in the hip severe, which sometimes extends into the knee and popliteal space. Venesection 8oz., which produced quite an impression on the pulse; lotion of Plumb. acetas and Mur. Ammon. to the thigh; give Calomel 2 grs., Ipecac 1-4 gr., Morph. 1-8 gr., every three hours.

11th, Coat removed from tongue, not so dry and swollen, but red and fiery looking; fever and delirium all night, had a remission this morning and sweat a little; has had no operation from the bowels since yesterday; the cold lotion to the thigh gave him much comfort. Continue the lotion and give Calomel 2 grs., Ipecac 1-4 gr. every three hours till five portions are taken, to be followed by a dose of *Oleum Ricini* should the bowels not act, and 3 grs. quinine every two hours, should a distinct remission occur in the morning.

12th. Has had three green looking operations since yesterday; tongue dry, red and crusted; sordes on teeth; delirium at times; abdomen tympanitic; pulse sharp, small and frequent; thigh and hip continues to swell, says he struck his hip against a rail on a fence, before he got sick: he is so delirious that I do not know what dependence to place in this assertion; the hip shows no bruise nor mark of any kind. Gave him *Hydrargyrum Cum Creta* 3 grs., Morph. 1-8 gr. every three hours, blister to abdomen, and cold lotions to thigh, and quinine should a distinct morning remission occur.

13th. He rested better last night, he is not so delirious, the lotion diminished the pain and suffering in the thigh, he cannot be moved without suffering great pain; keeps his knee and hip slightly flexed with the foot everted. The femoral vein can be felt like a hard cord under the finger, together with some smaller cord-like substances. The anterior part of the thigh is very tender and he cannot bear it touched; pulse 100 per minute and sharp; tongue red, dry and crusted; blister drawn well; tympanitis of bowels subsided; no discharge from the bowels since yesterday; sweats freely, and took the quinine in the morning remission. Prescribed Calomel 2 grs., Ipecac 1-4 gr. every two hours and lotion to the thigh.

14th. Had two operations from the bowels this morning of a vitiated bilious appearance, passed five *lumbrici*; pulse 100 and sharp; tongue dry, red, smooth and glossy; not so much delirium: pain in thigh severe; thigh hot, swollen and tender; leg begining to swell; he cannot be moved without suffering extreme pain. Gave Calomel 2 grs., Ipecac 1 gr., *Creta ppt.* 2 grs. every four hours, acetate of Ammonia as a diaphoretic; *Spigelia Marilandica* as an anthelmintic.

15th. Had several dark bilious stools, passed eight *lumbrici;* tongue not so red, still dry; sordes on teeth not so thick; pulse 100 per minute and sharp; thigh and leg swollen, painful and hot; the course of the veins can be easily traced from their cord-like feel. Continue lotion, and give 3 grs. Calomel and 1 gr. Ipecac every three hours.

16th. Much in the same condition as yesterday; no delirium; irritation of bowels diminishing. Continue the same treatment,

17th. Much the same; tongue inclined to scurf over, of a dry scaly appearance; pulse sharp, some symptoms of a mercurial impression being produced on the system; the pain in the thigh and groins not so great; complains of pain about the knee; right foot swelling. Give *Pulv. Dover* as an anodyne, and blue mass at night as an alterant.

From this, till the the 26th of the month, he appeared to improve in every respect, with the exception of the thigh and leg, which continued swollen and painful. It was more than double the other thigh; the skin was shining and glistening, and there was no pitting on pressure. He continued to take the blue mass and Dover powder; the pulse came down to 90, although it never lost its sharp beat. The discharges from his bowels became feculent and healthy looking, his appetite became good and craving; his whole body broke out in small pearly vesicles, (sudamina); his tongue lost its fiery appearance and assumed a pink healthy color and moist. On the 26th, a small discolored spot appeared to be sloughing on the sacrum, and another on the external maleolus of the right leg where it touched the bed, by his lying with his foot everted. He yet could not be removed, without suffering extreme anguish. The lead and ammonia lotion appeared to afford him greater comfort than any other local applications. He was blistered on the anterior part of the thigh, which relieved to some extent, the tenderness of the groin. Emaciation continued to proceed rapidly, although I endeavored to sustain his system by appropriate tonics and anodynes as circumstances demanded. On the 2d of October, the sore on the back was as large as a silver dollar and ulcerated to the depth of 1-4 of an inch, with hard elevated edges. The one on the external maleolus, was as large as a ten cent piece, and the foot was much swelled, pitting on pressure with the finger.—Groves' Liniment was applied to the bed sores, which consists of 2-3 dr. Castor Oil and 1-3 dr. Balsam Peru, which appeared to have a good effect on them and prevented further ulceration.

He continued much in the same condition, with good appetite and healthy, regular discharges from the bowels, complaining only of his leg and thigh, until the 9th, when hectic fever began to show itself in the afternoons, which would continue all night. The right foot begins to swell very much, the swelling extending up the calf of the leg, which pitted on pressure. He had also, a slight hacking cough, with slight expectoration of a very fœtid pus. On examination of the lungs with the stethescope, crepitant râles with a sonorous buzzing sound, were audible over the whole right lobes, particularly at the inferior portion: the morbid sounds were not so distinct in the left lobes of the lungs. The pain in the hip and thigh were mitigated to a considerable extent, although he could not be moved without excruciating torture.

On the 12th, the left foot began to swell, indicating effusion into it also; his appetite was good and bowels regular; he continued to cough and expectorate a fœtid pus: several more sloughing patches made their appearance on his back; the glans Penis inflamed and suppurating. I opened it with the lancet, it discharged a table-spoonful of yellow pus.

On the 15th, petechiäe, inky looking spots made their appearance on his fore arms. He complained of pain in the right arm and difficulty of using it, although it did not swell. Aphthae also appeared in the mouth. Suffice it to say, that the inferior extremities continued to swell, the skin shining and transparent, with pitting on pressure, indicating great anasarcous effusion; the petechiäe spread over his whole arms. He continued to cough up a very fœtid pus, which was very disagreeable to the attendants, and worn out with hectic, being greatly emaciated, and his feet and legs greatly swollen, on the last of the month, death terminated his sufferings.

His appetite was good, and his bowels regular till within three days of his death. I may state also that the temperature of the right foot was considerably below that of the left, for two weeks before his death, showing an impeded circulation, through the diseased veins of the right extremity.

Phlebitis generally occurs after injuries, surgical operations, &c., but in this case, no apparent cause was visible, he had enjoyed excellent health up to the period of the present attack. Dr. Robert Lee says, that from a number of cases and dissections, which he had made, crural Phlebitis is not peculiar to women who have been recently delivered, but that it may arise from other causes in the female, and that it has also occured in the male sex; the disease commencing either in the hemorrhoidal, vesical, or in some of the other branches of the internal Iliac veins in consequence of inflammation or organic changes of structure, in one or more of the pelvic viscerea, or in the superficial veins of the leg. Tweedie, Graves and Stokes, speak of it following fevers. In McNeff's case, some of his discharges were of a dysenteric character, and probably the disease had its origin in some of the hemorrhoidal veins; the hectic, disease of the lungs, and the general ulcerative disposition of his skin, when it was in contact with the bed, was the consequence of purulent matter getting into the circulation from the diseased veins. In some of Dr. Lee's cases, similar consequences, took place. He relates cases of destructive inflammation of the eyes, and also cough and fœtid expectoration, in connection with crural Phlebitis. The same also follows injuries and surgical operations.

